# Injury Related to Fall and Its Predictors among Medically Diagnosed Adults with Visual Impairment in Ethiopia: An Observational Cross-Sectional Study

**DOI:** 10.1155/2021/6686068

**Published:** 2021-02-27

**Authors:** Moges Gashaw, Biruk Adie Admass

**Affiliations:** ^1^Department of Physiotherapy, School of Medicine, College of Medicine and Health Sciences, University of Gondar Comprehensive Specialized Hospital, Gondar, Ethiopia; ^2^Department of Anesthesia, School of Medicine, College of Medicine and Health Sciences, University of Gondar Comprehensive Specialized Hospital, Gondar, Ethiopia

## Abstract

**Background:**

Fall-related injury is the common cause of unintentional injury and premature death among people with visual impairment. So far, the knowledge about fall-related injuries among medically diagnosed visual impairment people living in low- and middle-income countries is scarce. Hence, this study is a preliminary attempt to assess the fall-related injury and its determinants among adult people with medically diagnosed visual impairment individuals.

**Methods:**

An institutional-based cross-sectional study was conducted from March to July 2018 with a total sample size of 337 study participants. The study participants were recruited by using a systematic random sampling method. Univariable and multivariable binary logistic regression model analysis was used to identify predictors of fall-related injuries with IBM Statistical Package for Social Sciences version 23.

**Results:**

A total of 320 adults with visual impairments participants have participated in this study. The finding of this study was reported as follows: 24.7% of (95% CI: 20.0–29.4%) adults with visual impairments experienced one or more fall-related injuries. The main predictors of fall-related injuries identified by multivariate analysis were severity of visual impairment: moderate (AOR, 2.91; 95% CI: 1.23 – 6.87), severe (AOR, 3.58; 95% CI: 1.26–10.17), cause of visual impairment: cataract (AOR, 10.63; 95% CI: 2.49 – 45.26), diabetic retinopathy (AOR, 15.35; 95% CI: 2.51–93.96), taking medication (AOR, 6.35; 95% CI: 2.93–13.75), having family support (AOR, 2.13; 95% CI: 1.08 – 4.19), and depression (AOR 3.82, 95% CI: 1.27–11.45).

**Conclusion:**

Soft tissue injuries were the most common fall-related injuries reported by the study participants. The severity of visual impairment, the cause of visual impairment, taking one or more medication, having family support, and having depression were significant predictors of fall-related injuries.

## 1. Introduction

Visual impairments (VI) have an extreme impact on the physical, psychological, and socioeconomically development of individuals and societies [1]. Visual impairment is more prevalent in low- and middle-income countries (LMICs); about 80%–90% of the world's visually impaired people live in LMICs [2]. In Sub-Saharan African countries, including Ethiopia, visual impairment occurred on average for 70 million people. Visual impairment is reported to be an independent risk factor for fall-related injuries and fall-related death among older adults [3].

Falls are one of the most common leading causes of unintentional fall-related injury and a cause of premature death among people with visual impairment [4]. A fall is defined as an event which results in a person coming to rest inadvertently on the ground or floor or other lower level with/without fall-related injury [5]. Fall-related injury is the common cause of unintentional injury and premature death among people with visual impairment. In all regions of the world, fall-related death rates are the highest among people with visual impairment and adults over the age of 50 years [6, 7].

Falls can lead to moderate to severe injuries, fear of falling, loss of independence, and death. In 2012, the direct cost of falls adjusted for inflation was $30 billion; by 2020, the annual direct and indirect cost of fall-related injuries is expected to be doubled. Fall-related trauma accounted for 5.3% of all hospitalization of adults with high hospital charges [8]. Falls were the most common event with 54% of the participants with VI reporting at least one fall and 30% reported more fall and got fall-related injuries [9]. The prevalence of self-reported falls among people with visual impairment in Ethiopia was 26.8% [10].

The most common factors that may influence fall-related injuries and fall are age, sex, the severity of visual impairment, body mass index, psychosocial, physical environment, depression, decreased vestibular reflexes, decreased muscle strength, increasing postural sway, concurrent medical issues, fear of fall, and preexisting medical comorbidities directly increasing the incidence of falls [11–13].

Older adults with VI have one of the highest injury-related morbidity and mortality rates and have a poor prognosis and more complications after fall-related injuries, which have significant ramifications for associated health care costs. It has been demonstrated that the risk of unintentional injuries resulting from falls is higher for individuals with VI compared with those with normal vision [14]. Though, studies reported the burden of falls in different populations. So far, the knowledge about fall-related injuries among medically diagnosed VI people living in LMICs is scarce. Hence, this study is a preliminary attempt to assess the fall-related injury and its determinants among adult people with medically diagnosed visual impairment individuals. Thus, this evidence will help us to establish preventive measures among this population.

## 2. Methods

### 2.1. Study Design, Setting, and Participants

An institutional-based cross-sectional study was conducted from March to July 2019 among adults with medically diagnosed visual impairment during the study period. This eye care center provides all-inclusive clinical and community eye health services for twelve zones and serves as a major referral center for more than 14 million people living in and around the region, North West Ethiopia. It is estimated that a minimum of 5000 patients utilize different eye care services per year and this is the only clinical catchment area for visually impaired individuals in Gondar city, situated in the northern part of Ethiopia. Based on the 2016 population estimates of Gondar city administration bureau, Gondar had a total population of 335,000 with 3200/km^2^, with an estimated total household count of 53725 and 182000 (52%) women [15].

The study area is geographically a challenging mountainous landscape and unsafe sometimes even for normal-sighted people and perilous for liable people like older people, physically challenged people, and people with visual impairment. The study setting of the ophthalmic outpatient clinic receives a wide range of ambulatory VI patients, visiting for consultation, medication, eye-glasses, mobility aids, and surgery appointments, and the study setting is the only referral hospital for the entire Gondar region. Consequently, individuals with a wide spectrum of visual impairment visit this institution. As of 2017, the ophthalmic clinic cares for 40 to 60 visually impaired patients every day. So, an average of 1500 adult participants is likely to visit the clinic during the six weeks data collection period. Adults with visual impairment, who can walk independently with or without mobility aids and aged ≥18 years with no other hearing disability, were included in this study. However, adults with visual impairment who ambulate dependently in personal support and have a hearing impairment, which cannot communicate verbally and in writing, were excluded from the study.

### 2.2. Sample Size Determination and Sampling Procedure

The sample size required for this study was determined using the single population proportion formula [16] and calculated using the Epi Info software version 7.0 (Centres for Disease Control and Prevention, USA). The samples were obtained from a relatively small population (*N* = 1500).

The following assumptions were used to determine the required sample: prevalence of 50% since no past regional data exists, a confidence level of 95, 5% margin of error, and 5% margin of error. So, the sample was taken from a relatively small population (*N* = 1500), and the required sample size was obtained by the following calculation:

(1) $$\begin{array}{c} n=\frac{\left(za/2\right)2p\left(1-p\right)}{d^{2}}, \end{array}$$

where *n* = sample size, *Z* = 95% confidence limit (1.96), *p* = proportion of the population which take 50%, *d* = margin of error or degree of accuracy desired (0.05), and *n* = (1.96)2x (0.5) (0.5)/(0.05)^2^ = 385; since the sample were taken from a relatively small population, correction formula was used:

(2) $$\begin{array}{c} n=\frac{n}{1}+\frac{n}{N},\\ n=\frac{385}{1}+\frac{385}{1500}=\frac{385}{1.256}=306. \end{array}$$

The derived power calculated sample size was *n* = 306. Accounting for an estimated refusal or nonresponse rate of 10%, the final sample size was calculated to be *n* = 337.

The study participants were recruited by using a systematic random sampling method by arranging the patients based on their chart number from the registered engagement register selected in Kth interval each day during the study period. The first participant between 1 and K was selected randomly by a lottery method, and the next participant was interviewed every fourth interval. The route was continual until the sketchy eligible sample size was reached.

The operational definition of the variables: fall-related injury is any injury that results from a fall after diagnosis of vision problem. Did you have any injury as a result of a fall after knowing you are with a vision problem? (Yes/No). Visual impairment (in any eye): which could not be eliminated by refractive correction or lenses (noncorrectable); significant loss of vision on which Snellen's chart reading is less than 6/12 to no light perception; mild visual impairment, presenting with a visual acuity of less than 6/12(20/40) greater than or equal to 6/18(20/60); moderate visual impairment, presenting with visual acuity of less than 6/18(20/60) to greater than or equal to 6/60(20/200); and severe visual impairment, presenting with visual acuity of less than 6/60(20/200) to no light perception [17].

Physical activity was categorized as “Yes” or “No” based on the response to three questions: (1) Do you exercise regularly now? (2) If you exercise regularly, how many days a week do you exercise? (3) If you exercise regularly, how many minutes a day, on average, do you exercise? For question number 1, the response alternatives were “Yes” or “No” and for question no 2, the respondent has to fill the number of days/week, and for question no 3, the respondent has to fill the average minutes of exercising per day.

### 2.3. Study Procedures

After providing a verbal account and explaining about this study to the caregivers and the study participants, all study subjects signed the written informed consent statement. A structured data collection questionnaire (Additional File 1) was developed based on an all-embracing review of different literature [18–21]. The questionnaire was initially prepared in English and then translated into Amharic to the local language and back into English to check the consistency of the questions and corrections were made accordingly.

The questionnaire included domains like sociodemographic characteristics, behavioral characteristics, physical measurements, visual related characteristics, medical comorbidity, and fall-related characteristics. In addition, the medical charts of the participants were reviewed to extract additional information like the diagnosis of medical comorbidity, the type of medication the participants took, and the diagnosis of VI. Most of the study participants took antidepressant, antihypertensive medication for cardiovascular conditions and medication to control diabetes mellitus. The questionnaires were orally administered to each patient individually during an interview with four trained ophthalmic nurses and the responses of the patient were recorded in the data collection sheet. Factors related to visual impairment, visual acuity measurement, Snellen's *E*-chart measurement, and medical eye screening reports evaluated by an optometrist on the day of data collection were recorded by the data collector. Counterchecking of the daily filled questionnaire and regular supervision were done by MG and BA. To minimize the possible response bias, the questions were read aloud in a quiet ambiance following which the participants were told to repeat the question to assure reception of the question clearly. Care was taken by data collectors to simplify the questions as much as possible, accompany caregivers during self-reporting and explanations were given whenever question arose.

### 2.4. Statistics and Data Analysis

Data were entered using Epi-Info software version 7.1 and exported to the IBM Statistical Package for Social Sciences (SPSS) version 23 for the window for further analysis and for coding, recoding, storing, and further analysis. Descriptive statistics like frequencies, percentages, means, and standard deviations were used for all participant characteristics and factors associated with fall-related injuries. With fall-related injuries (categorized as Yes versus No) as a dependent variable, logistic regression analysis was done to determine the association with different independent variables.

Independent variables included in the regression models were sociodemographic characteristics (age, gender, residence, marital status, level of education, occupation, and income); physical measurements (height, weight, and BMI); behavioral factors (alcohol drink, smoking, and physical exercise); vision-related factors (mild vision impairment, moderate vision impairment, and severe vision impairment); and the current level of mobility, comorbidity, and drug intake. Multiple regression and interaction terms were employed to examine the potential association. Variables were entered into the model using forced entry and categories were used as covariates for detailed analyses. Results were considered statistically significant with 95% confidence intervals not containing unity (equal to *P* value < 0.05) for both the main effects and interaction terms.

Initially, bivariate analysis was conducted and independent variables that were found statistically significant were fitted into the multivariate model using the backward stepwise (likelihood ratio) method. We tested for potential statistically significant interactions by adding the product of the covariates in the multivariable-adjusted logistic regression models. Crude and adjusted odds ratios and 95% confidence intervals were calculated from univariate and multivariate logistic models for associations between the independent variables and the dependent variable. Variables were entered into the model using forced entry and categories were used as covariates for detailed analyses. Results were considered statistically significant with 95% confidence intervals not containing unity (*P* value < 0.05) for both the main effects and interaction terms.

When clear subgroups seemed to be present in the dataset, significance testing (Pearson *χ*2) and, if appropriately sized subgroups per category remained, logistic regression analyses were conducted. Finally, this study was reported in accordance with the STROBE guidelines (Additional File 2).

## 3. Results

### 3.1. Sociodemographic Characteristics of the Participants

A total of 320 adults with visual impairments participants were included in this study. This is a 94.9% response rate and beyond the power calculated sample size (*n* = 306). The reasons for nonresponses were no time (*n* = 7), not interested and refusal to participate (*n* = 6), and incomplete medical review chart profile (*n* = 4). The age of the study participants ranged from 22 to 83 years with a mean age of 55.94 ± 14.2 years. The mean body mass indexes of the participants were 19.97 kg/m^2^ (±2.2). Nearly two-thirds of the participant 70.3% had a normal range of BMI (18.5 to 24.9). Nearly, one-third (29, 4%) of the participants were housewives, most of them reported their religious affiliation as orthodox Christians (90.3%), and almost all of them (95.3%) reported no smoking habits. The sociodemographic characteristics of the participants are presented in Table 1.

### 3.2. Vision-Related Characteristics of the Study Participants

Among the study participants, nearly half (46.6%) of the participants were diagnosed with glaucoma, and more than one-third of adults had mild visual impairment (40.6%) and moderate VI (40.3%) based on Snell's E-chart reading.  Table 2 shows the vision-related characteristics of adults with visual impairments.

### 3.3. Fall-Related Injuries among Medically Diagnosed Adults with Visual Impairments

**Figure 1:**
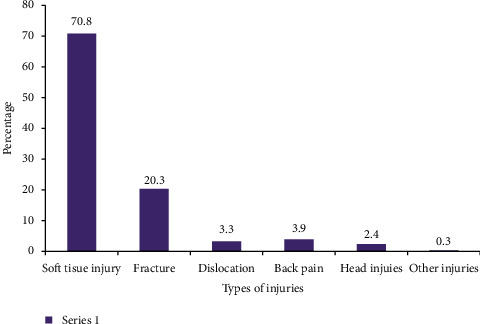
Types of fall-related injuries reported by adults with visual impairment in Gondar, Ethiopia (*n* = 79).

The predictors and distribution of fall-related injuries among medically diagnosed adults with visual impairments are shown in Table 3. In this study, (24.7%, 95% CI: 20.0–29.4%) people with visual impairment have sustained fall-related injuries. The higher fall-related injuries were noted among male participants (54.4%) and older adults (45.6%). Nearly, two-thirds (67.1%) of adults who noted fall-related injuries were diagnosed with glaucoma followed by cataract (26.6%). The types of fall-related injuries reported by the participants are shown in Figure 1.

### 3.4. Regression Analysis

In the univariate regression analyses, fall-related injuries were significantly associated with age, BMI, associated medical comorbidity, severity of VI, cause of VI, taking medication, family support, and depression. However, the multivariable analysis revealed that severity of visual impairment, cause of visual impairment, taking one or more medications, good family support, and having depression were significant predictors for fall-related injuries.  Table 4 shows the association between sociodemographic variables, vision-related variables, behavioral variables, and medical-related variables with dependent variables among the study participants.

## 4. Discussion

The finding of this study reported that 24.7% with (95% CI: 20.0–29.4%) adults with visual impairments experienced one or more fall-related injuries. This shows that fall-related injuries are the main reason for hospital visits; and one of the main causes of disability among visually impaired adults, in addition to having a socioeconomic impact on an individual and on the development of the countries [7, 9]. Among the study participants who have experienced fall-related injuries, 70.8% had soft tissue injuries which are bruising, skin damage, and abrasion, and 20.3% had reported fractures in different regions of the body. This finding is harmonized with the studies conducted in the USA [22]. The severity of visual impairment, cause of visual impairment, taking one or more medications, having family support, and having depression were significant predictors for fall-related injuries among visually impaired individuals.

The incidence of fall-related injures found in the current study is comparable with the results of the studies done in the United Kingdom 21% [23] and Suburban, Australia 26% [24]. However, the reported incidence of fall-related injuries in this study was found to be lower (24.7%) compared to the studies done in Sydney, Australia 63% [9]. The variation might be due to the difference in study participants; most of the study participants had been diagnosed with different eye disease including cataract, glaucoma, age-related maculopathy, diabetic retinopathy, uncorrected refractive error, and different other eye diseases, and most of the study participants were diagnosed with glaucoma (46.6%). However, all of the study participants of the studies of Australia included only patients with age-related maculopathy [9]. Another possible reason could be the difference in study design and sample size variation; this study is conducted in a large sample size with an institutional-based cross-sectional study design while the study done in Sydney, Australia, used seventy-six community dwelling diagnosed with age-related macular degeneration with community-based study conducted.

However, the incidence rate of fall-related injuries reported in this study was found to be higher when compared to the study conducted in the USA 10% [6]. The possible reason for this discrepancy observed in the incidence rate of fall-related injuries could be due to the difference in the study area, study participants, sample size, and study settings. Furthermore, factors like differences in the educational level of the participants, availability of mobility aids, awareness program on risk factors and preventions, adjunct care in the eye clinic, and health-related facilities might also be the reasons for the lower incidence of fall-related injuries in these countries.

Another remarkable finding in the present study was the severity of VI, the cause of VI, taking medication, having family support, and depression as independent predictors of fall-related injuries with higher odds. Those participants diagnosed with a moderate level of VI were 2.91 times and a severe level of VI 3.58 times more likely to have fall-related injuries as compared to individuals who had mild VI (Table 4). This result was supported by other studies [24–26]. The adjusted odds of fall-related injuries were 10.63 times higher among adults diagnosed with cataract and 15.35 times higher among visual impairment adults diagnosed with diabetic retinopathy as compared to other eye diseases. This finding is supported by different studies. People with glaucoma and diabetic retinopathy can have compromised visual field which is associated with poor postural stability and a greater capacity to bump into objects, which could also lead to a greater tendency to fall and fall-related injury [4, 27].

Adult people with visual impairment who had associated depression comorbidity were 3.82 times more likely to have fall-related injuries as compared to their counterparts in this finding. Evidence that depression contributes to fall risk is consistent with the documented association between depression and disability and depressive symptoms were associated with a twofold risk of an adverse fall event and unintentional injuries during the patient's episode of care [7, 28]. On the other hand, 87.5% of adults with visual impairment who had good family/social support are less likely to have fall-related injuries than counterparts of those not having social or family support.

This study also showed that taking medication was also found to be one of the predictors of fall-related injuries among medically diagnosed visually impaired adults in this study. The odds of having falls were 6.35 times higher among people who had taken one or more medications as compared to those individuals not taking medication. This finding was supported by different studies, which found that visually impaired people who had to take greater than three medications for different comorbidity were two times more likely to experience fall and fall-related injuries than counterparts. Different studies found that the total number of medications dispensed to the patient was significantly associated with fall and related injuries among older adults. This is due to the adverse effect of the medication, drug-drug interaction, and the metabolic effect of the drug on the body. Sedatives, antipsychotics, and sedating antidepressants cause drowsiness and slow reaction times. Some antidepressants and antipsychotics also cause orthostatic hypotension [22, 29]. The possible explanation might be medication use is a factor associated with falls and injuries in older adults. Polypharmacy, particularly the use of four or more medications, increases the risk of falling and related injuries [29]. Additionally, the use of certain classes of medications, including sedatives, hypnotics, antipsychotics, antidepressants, and antiarrhythmics, has demonstrated an increased association with falls [30, 31]. Although the use of medications may predispose VI older adults to falls and related injuries, medications may also cause changes in vision or vision loss, another risk factor for fall-related injuries in older adults [18, 32]. Many medications can lead to an ocular adverse event [33, 34]. Drugs that possess anticholinergic properties, such as antihistamines, antipsychotics, and tricyclic antidepressants, are associated with visual disturbances including blurred vision, diplopia (double vision), and cycloplegia (loss of accommodation). Amiodarone is a commonly used antiarrhythmic agent that can cause dose-dependent corneal deposits that may lead to lens opacities and halo vision.

## 5. The Strength and Limitation of the Study

The possible strength of this study was the inclusiveness of different types of study participants which were adults diagnosed with different types of eye disease; and all the measurements were undergone by ophthalmic nurses professional in order to exclude measurement bias. For the benefits of future research, there are some notable limitations, these fall-related injuries were self-reported and reviewed from patient's medical registration and identification charts. Another possible limitation, the cross-sectional nature of these studies, presents limitations in terms of casual association interpretation and not showing cause and effect.

## 6. Conclusion

Soft tissue injuries were the most common fall-related injuries reported by the study participants followed by fractures. Fall-related injuries were the main reason for hospital visits among adults with visual impairments. The severity of visual impairment, the cause of visual impairment, taking one or more medications, having family support, and having depression were significant predictors for fall-related injuries.

## Figures and Tables

**Table 1:** The sociodemographic characteristics of the study participants, Gondar, Ethiopia (*n* = 320).

Variables	Categories	Frequency (*n*)	Percent (%)
Age in years (mean age ((55.94 ± 14.2))	22–44	79	24.6
	45–64	132	41.3
	Greater than 64	109	34.1
Sex	Male	187	58.4
	Female	133	41.6
Residence	Urban	129	40.3
	Rural	191	59.7
Marital status	Not married	12	3.8
	Married	256	80.0
	Divorced	29	9.1
	Windowed	23	7.2
Religion	Orthodox Christian	289	90.3
	Protestant	2	0.6
	Muslim	29	9.1
Occupation	Housewife	94	29.4
	Farmer	142	44.4
	Civil servant	30	9.4
	Merchant	31	9.7
	Unemployed	7	2.2
	Retired	15	4.7
	Student	1	0.3
BMI (kg/m2) (mean 19.97 kg/m^2^ (±2.2)	Underweight (<18.5)	82	25.6
	Normal (18.5–24.9)	225	70.3
	Overweight (25–29.9)	13	4.1
	Obese (>29.9)	0.0	0.0
Income (ETB/month)	<1000	137	42.8
	1000–2000	99	30.9
	2001–3000	46	14.4
	>3000	38	11.9
Level of education	No formal school	189	59.1
	Primary school	67	20.9
	Secondary school	28	8.8
	Diploma	17	5.3
	Degree and above	19	5.9
Smoking habit	Never	305	95.3
	Past smoker	5	1.6
	Current smoker	10	3.1
Drinking alcohol habit	Never	194	60.6
	Past alcoholic	37	11.6
	Current alcoholic	89	27.8
Physical exercise	No	277	86.6
	Yes	43	13.6
Taking medication	No	218	68.1
	Yes	102	31.9
Types of medication (*n*=102)	Antihypertensive	25	24.5
	Psychotropic medication	6	5.9
	Diabetic medication	19	18.6
	Medication for different eye diseases	41	40.2
	Unspecified	11	10.8
Number of medications/day (*N*=102)	One	76	74.5
	Two	19	18.6
	Three and more	7	6.9

**Table 2:** Vision-related characteristics of the study participant in Gondar, Ethiopia (*n* = 320).

Variables	Categories	Frequency (*n*)	Percent (%)
Cause of visual impairment	Cataract	34	10.6
	Glaucoma	149	46.6
	ARM	22	6.9
	Diabetic retinopathy	12	3.8
	URE	43	13.4
	Other eye diseases	60	18.8
Severity of visual impairment	Mild visual impairment	130	40.6
	Moderate visual impairment	129	40.3
	Severe visual impairment	61	19.1

ARM : age-related maculopathy, URE : uncorrected refractive error.

**Table 3:** Fall-related injuries among medically diagnosed adults with visual impairments, Gondar, Ethiopia (*n* = 320).

Variables	Categories	Fall-related injury			
		No (%)	Yes (%)	*χ*2	*P*
Age in years (mean age ((55.94 ± 14.2))	22–44	65 (82.3)	14 (17.7)	6.64	0.35
	45–64	103 (78.0)	29 (22.0)		
	Greater than 64	73 (67.0)	36 (33.0)		
Sex	Male	144 (77.0)	43 (23.0)	0.69	0.41
	Female	97 (72.9)	36 (27.1)		
Residence	Urban	91 (70.5)	38 (29.5)	2.65	0.10
	Rural	150 (78.5)	41 (21.5)		
Marital status	Not married	9 (75.0)	3 (25.0)	17.6	0.001
	Married	201 (78.5)	55 (21.5)		
	Divorced	22 (75.9)	7 (24.1)		
	Windowed	9 (39.1)	14 (60.9)		
Religion	Orthodox Christian	220 (76.1)	69 (23.9)	2.25	0.32
	Protestant	2 (100.0)	0 (0.00)		
	Muslim	19 (65.5)	10 (34.5)		
Occupation	Housewife	67 (71.3)	27 (28.7)	15.97	0.01
	Farmer	114 (80.3)	28 (19.7)		
	Civil servant	27 (90.0)	3 (10.0)		
	Merchant	16 (51.6)	15 (48.4)		
	Unemployed	5 (71.4)	2 (28.6)		
	Retired	11 (73.3)	4 (26.7)		
	Student	1 (100.0)	0 (0.00)		
BMI (kg/m^2^) (mean 19.97 kg/m^2^ (±2.2)	Underweight (<18.5)	57 (69.5)	25 (30.5)	6.07	0.48
	Normal (18.5–24.9)	177 (78.7)	48 (21.3)		
	Overweight (25–29.9)	7 (53.8)	6 (46.2)		
Income (ETB/month)	<1000	102 (74.5)	35 (25.5)	0.77	0.86
	1000–2000	74 (74.7)	25 (25.3)		
	2001–3000	37 (80.4)	9 (19.6)		
	>3000	28 (73.7)	10 (26.3)		
Cause of visual impairment	Cataract	13 (38.2)	21 (61.8)	57.35	0.001
	Glaucoma	116 (77.9)	53 (22.1)		
	ARM	15 (68.2)	7 (31.8)		
	Diabetic retinopathy	3 (25.0)	9 (75.0)		
	Uncorrected refractive error	41 (95.3)	2 (4.7)		
	Other eye diseases	53 (88.3)	7 (11.7)	35.48	0.001
Severity of visual impairment	Mild	117 (90.0)	13 (10.0)		
	Moderate	93 (72.1)	36 (27.9)		
	Severe	31 (50.8)	30 (49.2)		
Smoking habit	Never	23 (75.7)	74 (24.3)	3.5	0.17
	Past smoker	2 (40.0)	3 (60.0)		
	Current smoker	8 (80.0)	2 (20.0)		
Drinking alcohol habit	Never	138 (71.1)	56 (28.9)	5.67	0.05
	Past alcoholic	28 (75.7)	9 (24.3)		
	Current alcoholic	75 (84.3)	14 (15.7)		
Taking medication	No	190 (87.2)	28 (12.8)	51.6	0.001
	Yes	51 (50.0)	51 (50.0)		
Physical exercise	No	206 (74.4)	71 (25.6)	0.99	0.32
	Yes	35 (81.4)	8 (18.6)		
Level of mobility	Independent without mobility aid	212 (81.2)	49 (18.8)	28.04	0.001
	Independent with mobility aid	29 (48.3)	30 (51.7)		
Family/social support	No	82 (66.7)	41 (33.3)	8.03	0.005
	Yes	159 (80.7)	38 (19.3)		
Medical comorbidity	No	173 (84.0)	33 (16.0)	23.36	0.001
	Yes	68 (59.6)	46 (40.4)		
Poor urine control	No	235 (75.8)	75 (24.2)	1.3	0.25
	Yes	6 (60.0)	4 (40.0)		

**Table 4:** Factors associated with fall-related injuries in adults with medically diagnosed visual impairment at Gondar, Ethiopia (*n* = 320).

Variables	Categories	Univariate COR (95% CI)	*P* value	Multivariate AOR (95%CI)	*P* value
Age in year	22–44	Ref.	—	Ref.	—
	45–64	1.31 (0.64–2.66)	0.46	0.58 (0.20–1.63)	0.30
	>64	2.29 (1.14–4.62)^*∗*^	0.02	0.52 (0.18–1.55)	0.24
BMI (kg/m^2^)	Underweight (<18.5)	Ref.	—	Ref.	—
	Normal (18.5–24.9)	0.62 (0.35–1.09)^*∗*^	0.09	0.78 (0.37–1.64)	0.50
	Overweight (25–29.9)	1.95 (0.59–6.41)	0.27	1.10 (0.24–5.14)	0.89
Cause of VI	Cataract	12.23 (4.28–34.9)^*∗*^	0.001	10.63 (2.49–45.26)^*∗*^	**0.001**
	Glaucoma	2.15 (0.89–5.18)^*∗*^	0.09	2,51 (0.78–8.13)	0.12
	ARM	3,53 (1.07–11.66)^*∗*^	0.03	3.4 (0.69–16.84)	0.13
	Diabetic retinopathy	22.7 (4.93–104.47)^*∗*^	0.001	15.35 (2.51–93.96)^*∗*^	**0.001**
	Uncorrected refractive error	0.36 (0.07–1.87)	0.22	0.92 (0.15–5.49)	0.93
	Other eye diseases	Ref.	—	Ref.	—
Severity of VI	Mild	Ref.	—	Ref.	—
	Moderate	3.48 (1.75–6.95)^*∗*^	0.001	2.91 (1.23–6.87)^*∗*^	**0.01**
	Sever	8.71 (4.07–18.66)^*∗*^	0.001	3.58 (1.26–10.17)^*∗*^	**0.02**
Medical comorbidity	No	Ref.	—	Ref.	—
	Yes	3.55 (2.09–6.01)^*∗*^	0.001	1.21 (0.57–2.57)	0.62
Taking medication	No	Ref.	—	Ref.	—
	Yes	7.14 (4.09–12.47)^*∗*^	0.001	6.35 (2.93–13.75)^*∗*^	**0.001**
Family support	No	2.09 (1.25–3.50)^*∗*^	0.005	2.13 (1.08–4.19)^*∗*^	**0.03**
	Yes	Ref.	—	Ref.	—
Depression	No	Ref.	—	Ref.	—
	Yes	3.74 (1.52–9.17)^*∗*^	0.004	3.82 (1.27–11.45)^*∗*^	**0.02**

^*∗*^Statically significant variables with *P* value <0.05; ref.: reference category; COR: crude odds ratio; AOR: adjusted odds ratio; CI: confidence interval; and VI: visual impairment.

## Data Availability

All data relevant to our findings are contained within the article. Further details on the dataset and queries will be made available upon reasonable request to the corresponding author (mogesgashaw1@gmail.com).

## Abbreviations

AOR: Adjusted odds ratio
ARM: Age-related maculopathy
BMI: Body mass index
CI: Confidence interval
COR: Crude odds ratio
LMICs: Low- and middle-income countries
SD: Standard deviation
USA: United State of America
URE: Uncorrected refractive error
VI: Visual impairment.

## Supplementary Materials

Supplementary Materials Additional File 1: English version questionnaire. Additional File 2: STROBE statement checklist.
