# Supplementary material for: Injury Related to Fall and Its Predictors among Medically Diagnosed Adults with Visual Impairment in Ethiopia: An Observational Cross-Sectional Study
Source: Adv Orthop. 2021 Feb 27;2021:6686068. doi: 10.1155/2021/6686068 (PMC7936889; doi:10.1155/2021/6686068)
Supplement: Supplementary Materials — Additional File 1: English version questionnaire. Additional File 2: STROBE statement checklist. [file 6686068.f1.zip › 6686068.f1/Additional file 2.docx]

Additional file2. STROBE checklist

**Title: Injury related to fall and its predictors among medically diagnosed adults with visual impairment in Ethiopia, Observational cross-sectional study.
STROBE** Statement—checklist of items that should be included in reports of **cross-sectional studies**

|  | Item No | Recommendation |
| --- | --- | --- |
| **Title and abstract** | 1 | 1. Study design is indicated in the abstract, methods section as school based cross-sectional study design. |
|  |  | (b) A balanced summary of what was done and what was found is provided in the methods and result section of the abstract |
| Introduction | | |
| Background/rationale | 2 | The scientific background and rationale for the investigation is reported. The rationale of the study was reported on the last paragraph of background section |
| Objectives | 3 | The general objectives of this study were stated as the final sentence of last paragraph under background session & the second sentence of the abstract. |
| Methods | | |
| Study design | 4 | It is presented in the first sentence of first paragraph of method section |
| Setting | 5 | A detailed description of the study area, location, and participant, described in the first and second paragraph of method section. |
| Participants | 6 | The study participants are clearly stated in the 2^nd^ and 3^rd^ paragraph of methods session. |
| Variables | 7 | Both outcome and predictor variables are mentioned study participant and statics sub-section in the methods in the last paragraph. |
| Data sources/ measurement | 8 | Source of data and data analysis methods are discussed in the 5^th^ and 6^th^ paragraphs of methods session |
| Bias | 9 | Efforts to address potential sources of bias were described in several part of method session. |
| Study size | 10 | Not relevant since it a survey study |
| Quantitative variables | 11 | All quantitative variables treated as qualitative after categorizing them in one of most commonly used categories. |
| Statistical methods | 12 | (*a*) Statistical methods used in this study are described under data statistics sub-section in the last part of method session. |
|  |  | (*b*) Both sub group analysis and interaction terms were used. |
|  |  | (*c*) There were no missing data in this study |
|  |  | *(d)* Not applicable |
|  |  | (*e*) Not applicable |
| Results | | |
| Participants | 13 | (a) Number of participants is presented in the first paragraph of results session and detail socio-demographic characteristics in the table 1. |
|  |  | (b) . Not applicable |
|  |  | (c) This was cross-sectional study so; there is no flow as that of longitudinal study. |
| Descriptive data | 14 | (a) Characteristics of study participants (e.g. demographic, painrelated variables,) and information on exposures and potential confounders is presented in tables 1 . |
|  |  | (b) There were no missing data in this study. |
| Outcome data | 15 | Outcome variable (fall related injury) described and summarized in table3. |
| Main results | 16 | (*a*) Unadjusted estimates and confounder-adjusted estimates and their precision (eg, 95% confidence interval) are presented in table 4. Discussed under regression analysis 1^st^ para in result section. |
|  |  | 1. Category boundaries of continuous variables were categorized and reported in all tables. |
|  |  | (*c*) Regression model was used and expressed in odds ratio in table 3. |
| Other analyses | 17 | No clear or significant sub group difference noted and interaction terms were used but non-significant. |
| Discussion | | |
| Key results | 18 | Key results to study objectives are discussed under discussion session with references. |
| Limitations | 19 | Limitations and possible strengths related to the current study are discussed in the strength and limitation session on the way of viewing direction for researchers. |
| Interpretation | 20 | A cautious overall interpretation of results considering objectives, results from similar studies, and other relevant evidence is discussed under limitation of discussion session. |
| Generalisability | 21 | Generalisability (external validity) of the study results are mentioned under conclusion section |
| Other information | | |
| Funding | 22 | Information regarding the source of funding (authors) and the role of the funders for the present study is presented under acknowledgment and funding section. |
